# Robot-assisted tumorectomy for an unusual pelvic retroperitoneal leiomyoma: A case report

**DOI:** 10.1097/MD.0000000000029650

**Published:** 2022-08-05

**Authors:** Zhe Zhang, Feiyu Shi, Junjun She

**Affiliations:** a Department of General Surgery, The First Affiliated Hospital of Xi’an Jiaotong University; b Center for Gut Microbiome Research, Med-X Institute, The First Affiliated Hospital of Xi’an Jiaotong University; c Department of High Talent, The First Affiliated Hospital of Xi’an Jiaotong University, Xi’an, Shaanxi, People’s Republic of China.

**Keywords:** case report, pelvic retroperitoneal leiomyoma, robotic surgery

## Abstract

**Rationale:**

Extrauterine leiomyoma occasionally occurs in rare locations with unusual growth patterns, especially pelvic retroperitoneal leiomyoma, which brings great challenges for surgeons to make a diagnosis. It is essential to distinguish benign from malignant retroperitoneal neoplasms according to the imaging manifestations. Laparotomy and laparoscopy are the common options for pelvic retroperitoneal neoplasms, while they may cause side effects during operation such as secondary damage. Appropriate surgical techniques should be adopted to ensure the complete excision of neoplasms meanwhile preserve the urination, defecation, and sexual function.

**Patient concerns:**

A 30-year-old woman was referred to our hospital because of dull pain in the perianal region for 1 month. Laboratory results including tumor markers were all within normal limits. The digital rectal examination revealed a huge and tough mass with smooth mucosa protruding into the rectal cavity from the rear area of rectum.

**Diagnosis:**

Imaging examinations were performed. Contrasted computed tomography (CT) of pelvis showed an enhanced retroperitoneal solid mass in the space between sacrum and rectum, and very close to the levator ani muscle. The mass was about 11.0*8.0 cm in size. Computerized tomography angiography (CTA) showed the distal branches of bilateral internal iliac artery went into the mass. Endoscopic ultrasonography (US) showed the mass compressed the rectum, as well as a clear boundary to the rectal wall. A histopathologic examination confirmed the mass was a pelvic retroperitoneal leiomyoma.

**Interventions:**

The patient underwent an operative resection with da Vinci Si surgical system after routine preoperative preparation. Anorectal motility was weekly monitored postoperation. No additional adjuvant therapy was performed.

**Outcomes:**

The patient could walk after 1 day and defecate normally on the third day after operation. She was discharged on the seventh postoperative day. No adverse events including pelvic floor hernia or defecation dysfunction occurred in the follow-up period. At 4 weeks follow-up, the patient was pain-free and recovered well.

**Lessons:**

Although imaging examinations were crucial for retroperitoneal neoplasms, histopathological examination remains the “gold standard” for making a definite diagnosis. This case highlights the possibility of retroperitoneal leiomyoma occurring in a woman of reproductive age and the advantages of robotic surgical system in pelvic retroperitoneal surgeries.

## 1. Introduction

Leiomyomas represent one of the most common tumors in women.^[1]^ According to epidemiology studies, leiomyomas occur in uterus in more than 70% of women.^[2,3]^ However, extrauterine leiomyomas located in retroperitoneum are rare, which may lead to misdiagnosis. Sarcoma represents the most retroperitoneal neoplasms, while retroperitoneum is one of the most common primary sites of sarcoma.^[4,5]^ While imagings are able to provide detailed information about the neoplasm and nearby neurovascular landmarks, histopathology remains the gold standard for definitive diagnosis.^[6]^ Appropriate surgical techniques are crucial for improving effects and recovery. We herein report a case of a 30-year-old woman diagnosed with pelvic retroperitoneal leiomyoma, which was a common tumor located in a rare position. We are reporting the application of robotic surgical system in the evaluation of such condition.

## 2. Case Presentation

**Figure 1.**
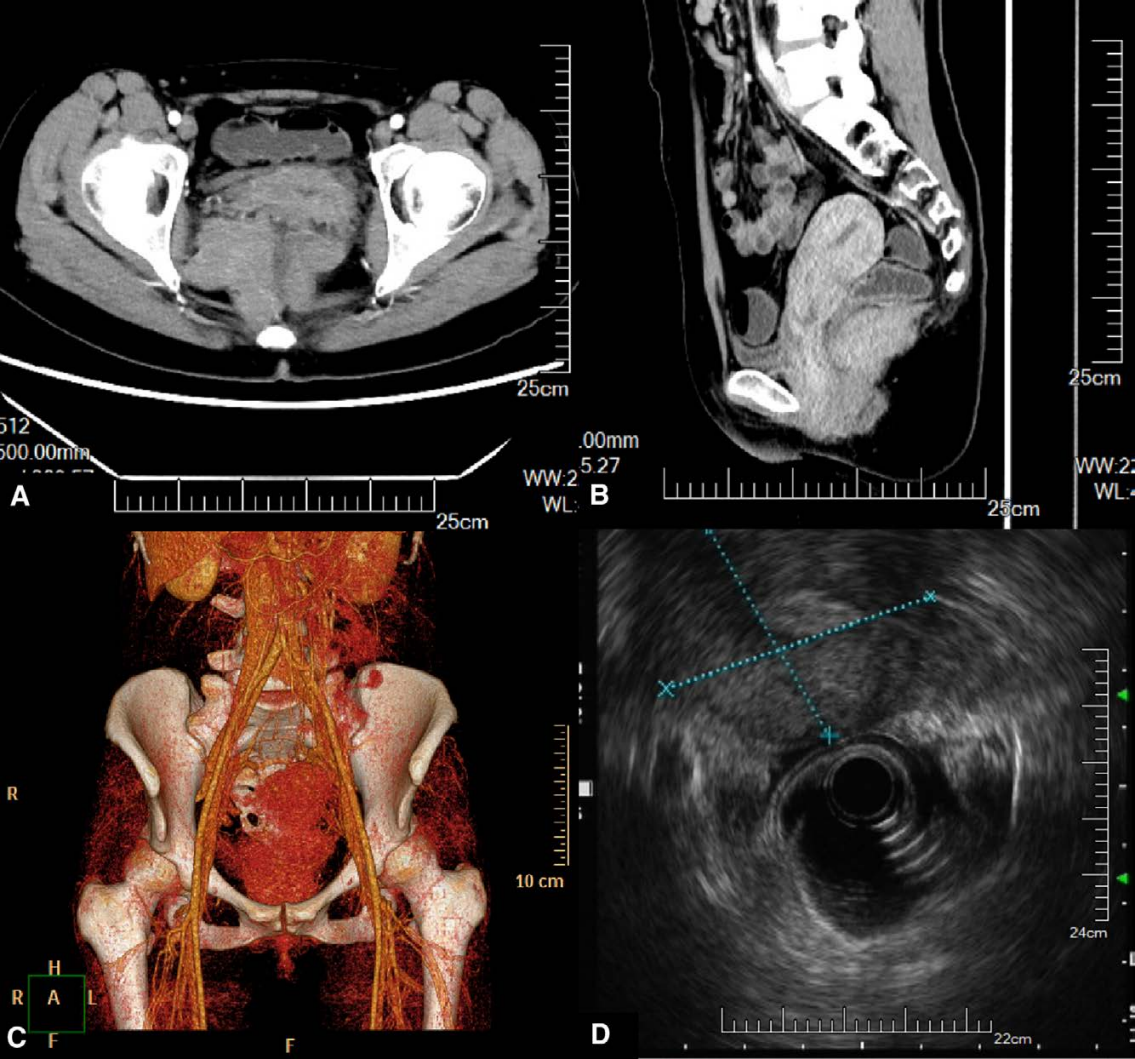
Image findings. A: Computed Tomography shows the mass very close to the levator ani muscle. B: The pararectal mass appeared to be homogeneous in density and slight enhancement in the arterial phase. C: The distal branches of bilateral internal iliac artery went into the mass. D: Endoscopic ultrasonography showed the well-defined mass with a homogeneous echotexture compressed the rectum, as well as a clear boundary to the rectal wall.

A 30-year-old Chinese woman was referred to our hospital because of dull pain in the perianal region for 1 month. The patient has repeated dull pain in the perianal region in the past 1 month. The discomfort aggravated along with the different body postures such as a sitting position or squatting position. However, she reported no urinary or gynecological disorders. There were no changes in bowel habits as well. She denied any fever, melena, or hematochezia. The patient had no relevant previous medical history. The patient underwent a vaginal delivery 2 years ago. Her family history was unremarkable. The digital rectal examination revealed a tough and huge mass with smooth mucosa protruding into the rectal cavity from the rear area of rectum. The mass was slightly painful during manual mobilization. Laboratory measurements such as complete blood cell counts, liver function, renal function, electrolyte levels, coagulation factors, and tumor markers were all within normal limits. Computed tomography (CT) of pelvis showed the mass whose size was about 11.0 × 8.0 cm was located in the space between sacrum and rectum, and very close to the levator ani muscle (Fig. 1A). The pararectal mass appeared to be homogeneous in density and slight enhancement in the arterial phase without necrosis or calcification (Fig. 1B). Contrasted computed tomography (CTA) showed the distal branches of bilateral internal iliac artery went into the mass (Fig. 1C). Colonoscopy revealed the intact rectal macosa, while the endoscopic ultrasonography (US) showed the well-defined mass with a homogeneous echotexture compressed the rectum, as well as a clear boundary to the rectal wall (Fig. 1D).

**Figure 2.**
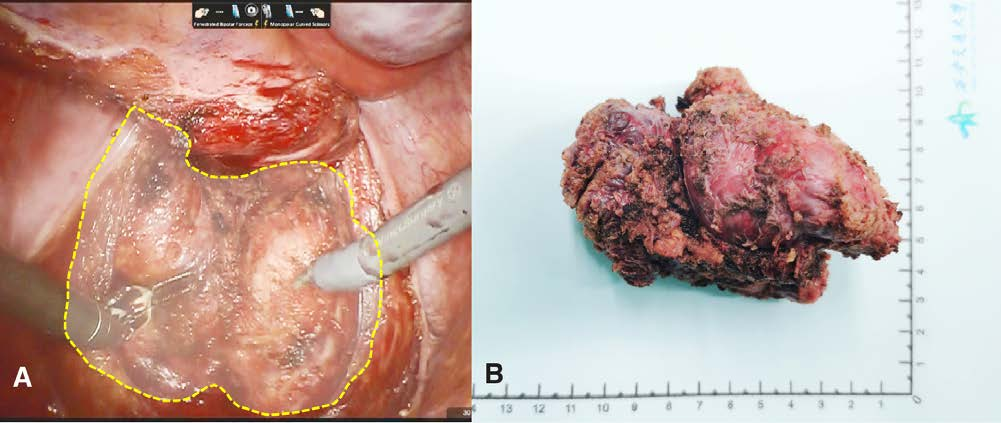
Surgical findings. A: The spindle-shaped mass located in the space between sacrum and rectum, adjacent to the levator ani muscle. B: Gross Appearance of Leiomyomas revealed the mass had an intact fibrous capsule measuring 11*8*7 cm.

Based on above evidence and descriptions, the mass was considered to be benign. The patient underwent an operative resection with da Vinci Si surgical system after routine preoperative preparation. As general anesthesia was induced, the patient was placed in a Lloyd Davis position on the operating table. After the pneumoperitoneum was established and the ports were placed, the operation began. During the examination, no obvious abnormality was found in pelvic cavity or organs. The peritoneum in peritoneal reflection between sacrum and rectum was incised. The rectum was lifted forward by a ribbon retractor to expose the retrorectal space. Then we dissected into the retrorectal plane between mesorectal fascia and prehypogastric nerve fascia and developed along until the level of the levator ani muscle was reached. The spindle-shaped mass was exposed to the surgical field, which has a clear boundary with the surroundings(Fig. 2A). We separate the tumor along the edge with the levator ani muscles, and a reddish-brown, elastic mass derived from pelvic floor muscle and had an intact fibrous capsule measuring 11 × 8 × 7 cm without hemorrhage in the central part was completely excised (Fig. 2B). The overall operative time was 270 minutes, while the robotic time and docking time was 210 minutes and 20 minutes separately. Intraoperatively blood loss was minimal, and no intraoperative complications occurred.

**Figure 3.**
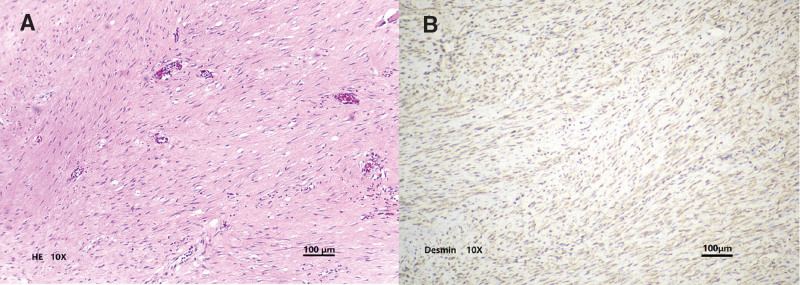
Microscopic examination. A: HE Staining of leiomyoma revealed a tumor composed of intersecting fascicles of typical smooth muscle cells. (HE: Hematoxylin and eosin, 10X). B: The tumor revealed strong positive staining for desmin (10X).

The final diagnosis was made by a histopathologic examination of resected specimens. Microscopic investigation revealed a tumor composed of intersecting fascicles of typical smooth muscle cells and the mitotic index was not high (Fig. 3A). No lymphocytes, plasma cells, or Russell body were present. Immunohistochemical staining showed tumor cells positive for desmin (Fig. 3B) and smooth muscle actin, as well as caldesmon, calponin, estrogen receptor (ER), and progesterone receptor (PR); but it showed negative for cluster of differentiation (CD) 34 and CD117, consistent with a diagnosis of pelvic retroperitoneal leiomyoma.

The patient could walk after 1 day and defecate normally on the third day after operation. She was discharged on the seventh postoperative day. Anorectal motility was weekly monitored. No adverse events including pelvic floor hernia or defecation dysfunction occurred in the follow-up period. At 4 weeks follow-up, the patient was pain-free and recovered well.

## 3. Discussion

Statistics show that the incidence rate of primary retroperitoneal tumors is 0.5–1.0/100,000, while most retroperitoneal tumors are malignant.^[7]^ However, pelvic retroperitoneal leiomyomas only constitute 1.2% of all primary retroperitoneal tumors.^[8]^ In a literature review, only 106 cases of retroperitoneal leiomyomas were reported from 1941 through 2008, and up to 40% of retroperitoneal cases are associated with synchronous or previously operated uterine myomas.^[9]^ Unusual growth patterns appear such as benign metastasizing leiomyoma, disseminated peritoneal leiomyoma, intravenous leiomyoma, parasitic leiomyoma, and retroperitoneal growth.^[10]^ Although extrauterine leiomyoma can be found in skin, respiratory system, digestive system, urinary system, or even circulatory system, they rarely have any histological malignant feature, including metastasizing.^[11–15]^ Therefore, isolated extrauterine leiomyomas are rare, which brings a great challenge for surgeons to make a diagnosis.

The typical patient of pelvic retroperitoneal leiomyoma is a female of reproductive age. Concerning their pathologic origin, pelvic retroperitoneal leiomyomas may arise from the hormonally sensitive smooth muscle elements or the embryonal remnants of müllerian or wolffian ducts.^[16–18]^ In our case, the patient in her early 30s did not receive a cesarean section or hysterectomy, and the mass was located in retroperitoneum. Therefore, uterine smooth muscle cells can’t seed in pelvic cavity, and the pelvic retroperitoneal leiomyomas may originate from vascular smooth muscle cells from the distal branches of bilateral internal iliac artery. A smooth muscle stem cell differentiates into a preclinical leiomyoma due to specific driver mutations, estrogen, and progesterone.^[19,20]^ WNT–β-catenin signaling pathway is involved in this process.^[21]^ Afterwards, with the help of the extracellular matrix, smooth muscle cells, vascular smooth muscle cells, fibroblasts, and fibroid-associated fibroblasts are stimulated to grow and therefore turn to clinical disease.^[18]^ Long-term exposure to risk factors including race, early menarche, delayed pregnancy, dietary, genetic alterations, and other than that obesity and parity, may also play a role in the pathogenesis.^[22–27]^ Further work such as single-cell sequencing is required to clarify the concrete mechanism that can lead to the initiation of primary retroperitoneal leiomyoma.

The most common clinical manifestation of retroperitoneal leiomyoma is pelvic mass palpation, though they differ in size, location, and amount. Symptoms are often related to compression of adjacent structures and can therefore cause gastrointestinal, urinary, and gynecological problems.^[28]^ Discomfort, fatigue, backache, and leg pain are usually nonspecific symptoms, hence pelvic retroperitoneal leiomyomas need to be differentiated from malignant retroperitoneal neoplasms. An abdominal or pelvic physical examination may reveal an enlarged tough mass.

Laboratory examinations sometimes help the clinicians make a diagnosis that cancer antigen 125 may be significantly elevated in patients’ plasma.^[29]^ However, US remains the first imaging examination to identify the solid mass owing to the benefits of lower cost. It provides accurate information about exact localization of the mass as well as the interactions between the mass and surrounding structures.^[30]^ Color Doppler might have the ability to show irregular vessels inside the mass clearly.^[31]^ Transanal or transvaginal US improves the sensitivity and specificity in the diagnosis of retroperitoneal neoplasms.^[32,33]^ Contrasted CT and magnetic resonance imaging (MRI) are considered as further examinations and they are highly accurate for differentiating benign from malignant, especially for the patients who have a large body mass index or had prior surgery.^[34–36]^ Pathological findings remain the “gold standard” for making a definite diagnosis. However, a diagnostic puncture may increase the risks of needle tract tumor cell seedings if the neoplasm is malignant in some cases.^[37]^

Surgical resection remains the mainstay of therapy for retroperitoneal leiomyoma.^[38]^ However, the approach to the surgery is largely empirical since no guideline has been published yet. An ideal surgical operation is to complete resection of the neoplasm and to preserve the integrity of the vessel and pelvic nerve. According to the previously reported cases, a laparotomy was the most chosen option, while the laparotomy would cause huge wounds and take a long time to recover.^[39]^ Laparoscopy is another option, the “chopstick effect” of the operation instruments, however, in the narrow pelvic space will increase difficulty in resecting leiomyoma because of its huge size and the adherence to adjacent structures (including the rectum, vagina, and pelvic plexus).^[40]^ Surgical robotic systems overcome limitations in laparoscopy such as the surgeon dexterity, sensory feedback, and visualization during operation.^[41]^ It can overcome the challenge of the narrow pelvic space and technically demanding dissection typical of pelvic and retroperitoneal surgery. It has also been proved that robotic surgery in pelvis and retroperitoneum has unique advantages over laparoscopy including less intraoperative bleeding, shorter hospital stay, and rapid postoperative recovery.^[42–44]^ In 2021, Crippa et al published a large retrospective cohort study and found although laparoscopic surgery was correlated to shorter operative time, robotic surgery was the most protective factor for odds to complications, which has lower transfusion requirements.^[45]^ In 2018, Prete et al published a meta-analysis compared robotic surgery with laparoscopic surgery in pelvic cavity.^[46]^ They found that the 2 groups shared equal overall short-term morbidity. However, Robotic surgery may be associated with lower conversion rate and longer operating time than laparoscopic approach. Similar conclusions have been obtained in gastrectomy and nephrectomy.^[47–49]^ Significant benefits of robotic surgery over laparoscopy have been demonstrated, while operative time and direct institutional cost may be the few disadvantages of robotic surgery.^[50]^ Robotic surgery is suitable for all kinds of benign disease in pelvic and retroperitoneal surgery regardless of medical costs, while invasion of major vessels is a relative contraindication for robotic surgery.^[51]^

## Author contributions

Conceptualization: Zhe Zhang, Junjun She.

Data curation: Zhe Zhang.

Investigation: Zhe Zhang, Feiyu Shi.

Funding acquisition: Junjun She.

Supervision: Junjun She.

Writing – original draft: Zhe Zhang, Feiyu Shi.

Writing – review & editing: Junjun She.
